# Population pharmacokinetics and Monte Carlo-based dosing optimization of trimethoprim-sulfamethoxazole

**DOI:** 10.1128/aac.00519-25

**Published:** 2025-09-03

**Authors:** Bo Chen, Yiying Chen, Ming Chen, Yunyi Mao, Yingbin Huang, Lili Zhou, Wenwei Wu, Xueyong Li, Xuemei Wu, Yu Cheng, Hongqiang Qiu

**Affiliations:** 1 Department of Pharmacy, Fujian Medical University Union Hospital 117890, Fuzhou, China; 2 College of Pharmacy, Fujian Medical University 74551, Fuzhou, China; 3 Department of Critical Care Medicine, Fujian Medical University Union Hospital 117890, , Fuzhou, China; Providence Portland Medical Center, Portland, Oregon, USA

**Keywords:** population pharmacokinetics, sulfamethoxazole, trimethoprim, Monte Carlo simulation, renal dysfunction, dose optimization

## Abstract

This study aimed to develop population pharmacokinetic (PopPK) models for intravenous sulfamethoxazole (SMX) and trimethoprim (TMP) to optimize dosing regimens for the treatment of *Pneumocystis jirovecii* pneumonia using these models. A prospective study was conducted in 79 patients treated with intravenous trimethoprim-sulfamethoxazole. PopPK models were developed using nonlinear mixed-effect modeling to evaluate the effects of liver function, kidney function, and genetic polymorphisms (*NAT2* and *CYP2C9*) on pharmacokinetic parameters. Monte Carlo simulations were employed to identify the optimal dosing regimen. Pharmacokinetic analysis of SMX and TMP included 232 post-dose plasma concentrations from 79 adult patients. A one-compartment model with first-order elimination best described the data. Creatinine clearance (CrCL) was significantly correlated with the pharmacokinetic parameters of both SMX and TMP, while continuous renal replacement therapy significantly influenced only the SMX model. Liver function, *NAT2*, and *CYP2C9* genotypes did not exhibit statistically significant effects on the models. Co-trimoxazole 50 mg/kg/day in a three-times-daily divided dose regimen is feasible for patients with CrCL of <15 mL/min. However, in patients with normal renal function, the guideline-recommended 90 mg/kg/day dose demonstrates a risk of supratherapeutic exposure. This study provides critical pharmacokinetic insights into SMX and TMP for patients, highlighting the necessity for dose adjustments in those with renal dysfunction. The currently recommended dosing regimens in clinical guidelines pose a risk of excessive drug exposure. Our study offers a more precise dosing strategy to optimize treatment efficacy and safety.

## INTRODUCTION

Co-trimoxazole, a broad-spectrum antibiotic combination of sulfamethoxazole (SMX) and trimethoprim (TMP), exerts its effect through synergistic inhibition of dihydrofolate synthesis and metabolic processes in pathogens. It demonstrates broad-spectrum efficacy against both gram-positive and gram-negative bacterial strains, with additional activity against atypical microorganisms, including *Nocardia* spp. and *Pneumocystis jirovecii* pneumonia (PCP) (1, 2). Despite the development of newer antibiotics that have largely replaced sulfonamides in treating many bacterial infections, co-trimoxazole maintains significant clinical relevance and remains the first-line therapeutic option for PCP (3).

Co-trimoxazole is clinically administered through both intravenous and oral formulations. The oral formulation demonstrates favorable absorption characteristics with bioavailability approaching 100%, achieving peak plasma concentrations within 2–3 h post-administration (4). SMX predominantly distributes within extracellular compartments, whereas TMP exhibits favorable tissue penetration characteristics, including a large apparent volume of distribution and broad tissue dissemination (5). Hepatic metabolism primarily converts SMX into inactive acetylated derivatives, whereas TMP undergoes limited hepatic processing (6). Although these metabolites lack antimicrobial efficacy, their progressive accumulation may induce concentration-dependent toxicities that contribute to co-trimoxazole’s adverse effect profile (7). Additionally, previous studies have demonstrated that patients with chronic liver disease exhibit significantly elevated plasma concentrations of SMX. Furthermore, a correlation has been observed between serum creatinine levels and TMP plasma concentrations (8). SMX hepatic metabolism primarily occurs through two pathways: *CYP2C9* hydroxylation and N-acetylation catalyzed by *NAT2* (4). A Japanese study demonstrated that *NAT2* genetic polymorphisms significantly influence SMX pharmacokinetics, with slow acetylators exhibiting a higher incidence of adverse reactions compared to rapid acetylators (9). Furthermore, emerging evidence indicates that mutations in the dihydrofolate reductase gene of PCP can confer resistance to co-trimoxazole (10). This might explain that suboptimal drug exposure may contribute to treatment failure against these resistant strains.

SMX and its metabolites are primarily excreted via the kidneys, with the majority eliminated unchanged through renal clearance and a minor fraction (<10%) excreted through biliary excretion (11). Both SMX and TMP exhibit renal function-dependent clearance, and impaired kidney function may lead to systemic accumulation of those agents. In patients undergoing continuous renal replacement therapy (CRRT), SMX clearance increases approximately 3.5-fold compared to non-CRRT patients (83.9 vs 24.4 mL/min) (12), posing significant dosing challenges. Substantial interindividual variability in serum drug concentrations persists even after dose normalization by body weight among PCP patients receiving high-dose therapy (13, 14). Reported adverse reactions include hypersensitivity reactions, hyperkalemia, gastrointestinal disturbances, systemic lupus erythematosus, hepatotoxicity, nephrotoxicity, and central nervous system toxicity (15–18). Therefore, therapeutic drug monitoring (TDM) is recommended for patients with hepatic or renal impairment receiving high-dose co-trimoxazole therapy (19).

Compared to other antibiotics, there is no established consensus regarding the optimal pharmacokinetic/pharmacodynamic (PK/PD) parameters for SMX and TMP, and the PK/PD indices most closely associated with bactericidal efficacy remain inconsistent. SMX and TMP demonstrate dual antimicrobial characteristics, exhibiting time-dependent bacteriostatic activity and concentration-dependent bactericidal activity against specific pathogens (20). For PCP treatment, therapeutic targets have been empirically established through clinical observations due to the technical challenges in cultivating PCP *in vitro* (21, 22). A clinical trial involving 11 PCP patients demonstrated that maintaining serum concentrations of SMX >100 mg/L and TMP >5 mg/L was associated with improved clinical outcomes (23). This finding was supported by another study of eight adult PCP patients, which identified TMP concentrations below 5.5 mg/L as a risk factor for treatment failure (14). However, therapeutic concentrations must be balanced against potential toxicity, with earlier overview and guidelines establishing toxicity-related thresholds at SMX >200 mg/L and TMP >10 mg/L (24, 25).

The establishment of optimal dosing regimens for intravenous administration to achieve therapeutic concentrations in PCP treatment remains challenging. Current evidence suggests that dosage adjustments are necessary for patients with renal impairment or those undergoing CRRT; however, the proposed dosing strategies lack robust validation through PopPK modeling studies (20, 26). Furthermore, existing population pharmacokinetic (PopPK) studies mainly focus on oral administration, with limited data available on intravenous administration. Additionally, current PopPK models have not adequately addressed the influence of hepatic function and genetic polymorphisms of metabolic enzymes on dosage optimization. Current guidelines, including those from the Food and Drug Administration (FDA), recommend a co-trimoxazole dosing regimen of 90–120 mg/kg/day (equivalent to SMX 75–100 mg/kg/day and TMP 15–20 mg/kg/day, respectively). The drug should be avoided in patients with creatinine clearance (CrCL) of <15 mL/min if possible since the SMX metabolites will accumulate to potentially toxic concentrations, while a 50% dose reduction is recommended for patients with CrCL of 15–30 mL/min.

Therefore, this study aims to develop a PopPK model using data from PCP patients receiving intravenous treatment, with the primary objective of characterizing the impact of hepatic and renal function on drug clearance. The study will systematically evaluate various covariates, including liver and kidney function parameters and metabolic enzyme genetic polymorphisms, to identify patient-specific factors contributing to interindividual variability. Based on the developed PopPK model, we aim to establish evidence-based dosing strategies optimized for specific patient populations.

## MATERIALS AND METHODS

### Patients

This prospective study was conducted at Fujian Medical University Union Hospital between March 2023 and October 2024, encompassing both intensive care units and general inpatient wards. The study criteria are summarized as follows: inclusion criteria include (i) patients aged ≥18 years with a confirmed diagnosis of PCP and (ii) patients receiving intravenous co-trimoxazole as part of their treatment; exclusion criteria include patients treated with hepatic enzyme inducers (e.g., carbamazepine and rifampin) during the study period.

Data were extracted from the hospital’s electronic medical records system, encompassing details on dosages, administration frequencies, plasma concentrations, and the use of CRRT. Demographic information, including sex, age, height, and weight, was also collected. Additionally, a comprehensive set of laboratory parameters was analyzed, including total bilirubin (TBIL), direct bilirubin (DBIL), total protein (TP), albumin (ALB), alanine aminotransferase (ALT), aspartate aminotransferase (AST), γ-glutamyl transferase (GGT), alkaline phosphatase (ALP), prothrombin time (PT), serum creatinine, and CrCL, the latter of which was calculated using the Cockcroft-Gault equation (27). Liver function was assessed and classified according to the Child-Pugh scoring system, which incorporates TBIL, ALB, PT, ascites, and hepatic encephalopathy (28).

### Sample collection and analysis

Therapeutic regimens for co-trimoxazole, including dosage and duration, were determined by a multidisciplinary clinical team. To ensure patient compliance and address ethical considerations, blood samples were collected at various time points following intravenous infusion, with each infusion time lasting approximately 1 h. Two to three samples were collected per patient: a peak concentration sample (immediately post-infusion), a trough concentration sample (pre-next dose), and/or an intermediate sample between peak and trough time points. Detailed sampling time points are outlined in Table 1.

**TABLE 1:** Sampling time points

Dosing frequency	Sampling times (h)^a^
Every 6 h	1, 3, and 6
Every 8 h	1, 4, and 8
Every 12 h	1, 6, and 12

^a^ Sampling times were measured relative to the initiation of intravenous infusion administration.

After collection, the blood samples were centrifuged at 9,380 × *g* for 5 min, and the supernatant obtained was stored at −80 ℃ until further analysis. Plasma concentrations of the analytes were quantified using a validated liquid chromatography-tandem mass spectrometry method (29). The calibration curves demonstrated acceptable linearity, with ranges of 3.12–400.0 mg/L for SMX and 0.20–25.0 mg/L for TMP. The method’s accuracy, as determined by intraday and interday studies, ranged from 92.9% to 112.6%, with a coefficient of variation between 4.3% and 9.7%. Additionally, the plasma stability and performance during freeze-thaw cycles met all analytical requirements, confirming the stability and reliability of the methodology.

Single-nucleotide polymorphism (SNP) loci were detected using multiplex polymerase chain reaction amplification. The *NAT2* gene harbors at least 13 SNPs. Genotyping three specific variants—341T>C (rs1799929), 590G>A (rs1799930), and 857G>A (rs1799931)—enables determination of haplotypes and diplotypes in most of the studied Asian population (30). These SNPs correspond to the five, six, and seven alleles, respectively, with the wild-type haplotype (no mutations) designated as NAT2*4. In Asian populations, the CYP2C9*3 allele is associated with reduced metabolic activity. The corresponding SNP is characterized by the 1075A>C variant (rs1057910), with CYP2C9*1 representing the wild-type allele lacking this mutation (31). Genomic DNA was extracted from blood samples using a commercial blood genomic DNA extraction kit (Sangong, Shanghai, China) following the manufacturer’s protocol. Specific primers were designed for PCR amplification, and the resulting PCR products were pooled in equimolar amounts. Sequencing was performed on the HiSeq XTen sequencer (Illumina, San Diego, CA, USA). SNP genotypes were determined using Samtools software (version 0.1.18), and haplotype configurations for each individual were inferred using RStudio (version 4.3.2). Based on the inferred haplotypes, individuals were classified into acetylator phenotypes: rapid metabolizer (homozygous wild-type individuals [NAT2*4/4, CYP2C9*1/*1]); intermediate metabolizer (individuals carrying one wild-type and one mutant haplotype [e.g., NAT2*4/*6 and CYP2C9*1/*3]); slow metabolizer (homozygous mutant individuals [e.g., NAT2*6/*6, NAT2*6/*7, and CYP2C9*3/*3]). This classification system enabled the stratification of individuals according to their *NAT2* and *CYP2C9* metabolic activity.

### Population pharmacokinetic analysis

PopPK analyzes were conducted using Phoenix NLME software (version 8.0; Pharsight, Mountain View, CA, USA). Model parameters were estimated through the first-order conditional estimation method with extended least squares. Interindividual variability was characterized using an exponential residual model, while intraindividual variability was accounted for through additive, proportional, and combined error structures. During the development of the structural model, both one- and two-compartment models with first-order elimination were systematically evaluated. Model selection was guided by the reduction in the objective function value, which was determined by comparing the difference in −2 log likelihood between nested models. Various demographic and clinical factors potentially influencing the pharmacokinetics of the two components were evaluated as covariates, including gender, age, height, weight, TBIL, DBIL, TP, ALB, ALT, AST, GGT, ALP, CrCL, *NAT2* genotypes, *CYP2C9* genotypes, and Child-Pugh classification. Continuous covariates were incorporated using linear, exponential, and power functions, whereas categorical covariates were implemented as power functions within the model.

The final model was evaluated using goodness-of-fit (GOF) plots and 1,000 prediction-corrected visual predictive checks (pc-VPC). GOF plots were used to assess the fit of the final model, including observed concentration vs population prediction concentration (PRED), individual population prediction concentration, and conditional weighted residuals vs time and PRED plots. The model parameters and their corresponding confidence intervals were evaluated through nonparametric bootstrapping with 1,000 resampled data sets.

### Monte Carlo simulation

Monte Carlo simulations using the final model were performed to evaluate optimal dosing regimens for treating PCP infection. Current guidelines recommend co-trimoxazole at 90–120 mg/kg/day, with dose reduction required for patients with renal impairment (20, 24, 32). Therefore, simulations incorporated CrCl values of <15, 15–29, 29–49, 50–79, and 80–120 mL/min, testing regimens including twice daily (BID), three times daily (TID), and four times daily dosing at 50, 55, 65, 70, and 90 mg/kg/day. Pharmacodynamic targets were established based on steady-state maximum plasma concentrations (*C*_max,ss_) during the dosing interval. The therapeutic window was defined with lower thresholds of 100 mg/L for SMX and 5 mg/L for TMP, while the upper safety limits were set at 200 mg/L for SMX and 10 mg/L for TMP (24).

## RESULTS

### Population

This study enrolled a total of 79 patients, from whom 232 plasma samples were collected for pharmacokinetic analysis. The demographic and clinical characteristics of the study population are summarized in Table 2. The cohort predominantly consisted of male patients (77.2%), with median values of 64 years for age and 60 kg for body weight. During the treatment period, CRRT was administered to 19 patients (24.1%). Renal function assessment revealed that 32 patients (40.5%) maintained normal renal function (CrCL ≥80 mL/min), while 48 patients (59.5%) exhibited renal dysfunction (CrCL <80 mL/min), with a median CrCL of 75.7 mL/min across the population. Based on the Child-Pugh classification system, patients were stratified into three hepatic function groups: Child-Pugh A (54 patients, 73.4%), Child-Pugh B (18 patients, 22.8%), and Child-Pugh C (7 patients, 8.9%). Furthermore, *NAT2* acetylator status analysis identified 30 patients (37.9%) as rapid acetylators and 68 patients (86.1%) as rapid metabolizers for *CYP2C9*.

**TABLE 2:** Clinical characteristics

Characteristics^*a*^	All patients
Sex (male/female), *n* (%)	61 (77.2)/18 (23.8)
Age (years)	64 (54–73)
Height (cm)	170.0 (165–175)
Weight (kg)	60.0 (55–70)
Continuous renal replacement therapy, *n*	19 (24.1)
Total bilirubin (μmol/L)	21.7 (10.6–36.5)
Direct bilirubin (μmol/L)	10.9 (5.4–27.2)
Total protein (g/L)	60.9 (54.0–68.5)
Albumin (g/L)	34.4 (30.8–37.3)
Alanine aminotransferase (U/L)	34.5 (17.0-56.0)
Aspartate aminotransferase (U/L)	32.0 (17.0–58.1)
Gamma-glutamyl transferase (U/L)	63.0 (26.4–107.3)
Alkaline phosphatase (U/L)	76.0 (57.0–105.3)
Serum creatinine (μmol/L)	77.8 (59.0–135.0)
CrCL^*b*^ (mL/min)	75.7 (51.0–93.7)
CrCL <80 mL/min	47 (59.5)
CrCL ≥80 mL/min	32 (40.5)
*NAT2* ^*c*^	
RA	30 (37.9)
IA	35 (44.4)
SA	14 (17.7)
*CYP2C9* ^*d*^	
NM	68 (86.1)
IM	7 (8.8)
PM	4 (5.1)
Child-Pugh classification^*e*^	
A	54 (68.3)
B	18 (22.8)
C	7 (8.9)

^*a*^ Data are expressed as median (range) or *n* (%).

^*b*^ CrCL calculated using the Cockroft-Gault equation.

^*c*^ *NAT2* phenotype classification: IA, individual acetylator; RA, rapid acetylator; SA, slow acetylator.

^*d*^ *CYP2C9* phenotype classification: IM, intermediate metabolizer; PM, poor metabolizer; RM, rapid metabolizer (31).

^*e*^ Child-Pugh calculated based on TBIL, ALB, PT, ascites, and hepatic encephalopathy.

### Population pharmacokinetic analysis

The PopPK characteristics of both SMX and TMP were best described by a one-compartment model with first-order elimination kinetics. For both models, residual variability was appropriately characterized using a proportional error model. The covariate selection process, including the evaluation of potential relationships between covariates and pharmacokinetic parameters, is detailed in Table 3 (for SMX) and Table 4 (for TMP). Covariate analysis revealed that CrCL significantly influenced the pharmacokinetics of both SMX and TMP, while CRRT was identified as a significant covariate exclusively for the SMX model. The final parameter estimates for the PopPK models, including fixed effects and random effects, are comprehensively presented in Table 5 (for SMX) and Table 6 (for TMP). The expression of the final SMX model is as follows:


(1) V=tv V×exp(ηV)

(2) $$\begin{array}{l} CL=tv\;CL\times (\frac{CrCL}{75.7}{)}^{dCLdCrCL}\times exp\;[dCLdCRRT\times (CRRT==1)]\;\times \exp\;(\eta CL). \end{array}$$

**TABLE 3:** SMX model selection and development^*a*^*^,^*^*b*^*^,c^*

Model	Model description	OFV	ΔOFV	*P* value
1	One-compartment model	2,091.68	–	–^*d*^
2	Two-compartment model	2,123.61	–	–
The choice of residual variability model (one-compartment model)				
1	Additive error model	2,091.68	–	–
3	Proportional error model	2,091.64	–	–
4	Combined error model	2,094.38	–	–
Forward inclusion				
3	Basic model	2,091.64	–	–
5	Add CRRT on CL in model 3.	2,064.79	−26.85	<0.001
6	Add CrCL on CL in model 5.	2,048.04	−16.75	<0.001
7	Add WT on CL in model 6.	2,040.52	−7.52	<0.01
Backward elimination				
8	Remove CRRT on CL in model 7.	2,064.88	24.36	<0.001
9	Remove CrCL on CL in model 7.	2,053.75	13.23	<0.001
10	Remove WT on CL in model 7.	2,047.09	6.57	<0.05

^*a*^ CL, clearance (L/h); CrCL, creatinine clearance; CRRT, continuous renal replacement therapy; OFV, objective function value; *V*, volume of distribution; WT, weight.

^*b*^ *P* value, a decrease in the OFV was referred to as the *χ*^2^ distributions to assess significance.

^*c*^ ΔOFV, the difference between the OFV of the covariate model and the basic model.

^*d*^ – indicates not applicable.

**TABLE 4:** TMP model selection and development^*a*^*^,^*^*b*^^,*c*^

Model	Model description	OFV	ΔOFV	*P* value
1	One-compartment model	559.47	–	–^*d*^
2	Two-compartment model	579.49	–	–
The choice of residual variability model (one-compartment model)				
1	Additive error model	559.47	–	–
3	Proportional error model	557.15	–	–
4	Combined error model	560.66	–	–
Forward inclusion				
3	Basic model	557.15	–	–
5	Add CrCL on CL in model 3.	542.83	−14.32	<0.001
6	Add WT on CL in model 5.	532.38	−10.48	<0.001
7	Add age on CL in model 6.	525.19	−7.19	<0.01
8	Add CRRT on CL in model 7.	521.07	−4.12	<0.05
Backward elimination				
9	Remove CrCL on CL in model 7.	539.43	18.36	<0.001
10	Remove WT on CL in model 7.	529.81	8.74	<0.05
11	Remove AGE on CL in model 7.	526.98	5.91	<0.01
12	Remove CRRT on CL in model 7.	524.99	3.92	<0.05

^*a*^ CL, clearance (L/h); CrCL, creatinine clearance; OFV, objective function value; *V*, volume of distribution; CRRT, continuous renal replacement therapy.

^*b*^ *P* value, a decrease in the OFV was referred to as the *χ*^2^ distributions to assess significance.

^*c*^ ΔOFV, the difference between the OFV of the covariate model and the basic model.

^*d*^ – indicates not applicable.

**TABLE 5:** Population pharmacokinetic model estimates and bootstrap results for SMX^*a*^

Parameters	Final model		Bootstrap	
	Estimate	RSE %	Median	95% CI
tv V (L/kg)	0.32	3.95	0.33	0.30–0.35
tv CL (L/kg/h)	0.02	5.0	0.02	0.18–0.22
dCLdCrCL	0.17	27.75	0.18	0.05–0.32
dCLdCRRT	0.59	15.81	0.61	0.41–0.78
Interindividual variability				
ω^2^ V	0.08	26.83	0.13	0.06–0.22
ω^2^ CL	0.16	3.91	0.17	0.08–0.25
Residual variability				
Proportional error	0.09	16.46	0.09	0.06–0.12

^*a*^ CI, confidence interval; CL, clearance of the central compartment; dCLdCrCL, fixed-parameter coefficient of creatinine CrCL to CL; dCLdCRRT; fixed-parameter coefficient of creatinine CRRT to CL; RSE, relative standard error; *V*, volume of distribution of the central compartment; *ω*^2^, variance of interindividual variability.

**TABLE 6:** Population pharmacokinetic model estimates and bootstrap results for TMP^*a*^

Parameters	Final model		Bootstrap	
	Estimate	RSE %	Median	95% CI
tv V (L/kg)	8.22	5.28	8.24	7.37–9.08
tv CL (L/kg/h)	0.11	5.55	0.1	0.05–0.21
dCLdCrCL	0.29	23.91	0.29	0.15–0.42
Interindividual variability				
ω^2^ V	0.19	16.78	0.18	0.13–0.25
ω^2^ CL	0.25	3.8	0.25	0.14–0.25
Residual variability				
Proportional error	0.27	7.84	0.06	0.05–0.07

^*a*^ CI, confidence interval; CL, clearance of the central compartment; dCLdCrCL, fixed-parameter coefficient of creatinine CrCL to CL; RSE, relative standard error; *V*, volume of distribution of the central compartment; *ω*^2^, variance of interindividual variability.

The expression of the final TMP model is as follows:


(3) V=tv V×exp(ηV)

(4) $$\begin{array}{l} CL=tv\;CL\times (\frac{CrCL}{75.7}{)}^{dCLdCrCL}\times exp\;(\eta CL). \end{array}$$

The term tv represents the typical value, while dCLdCrCL and dCLdCRRT are fixed-effect coefficients. CRRT indicates whether the patient is receiving continuous renal replacement therapy. *η* is a random variable assumed to follow a normal distribution with a mean of 0, and 75.7 represents the median CrCL value.

### Model validation

**Fig 1:**
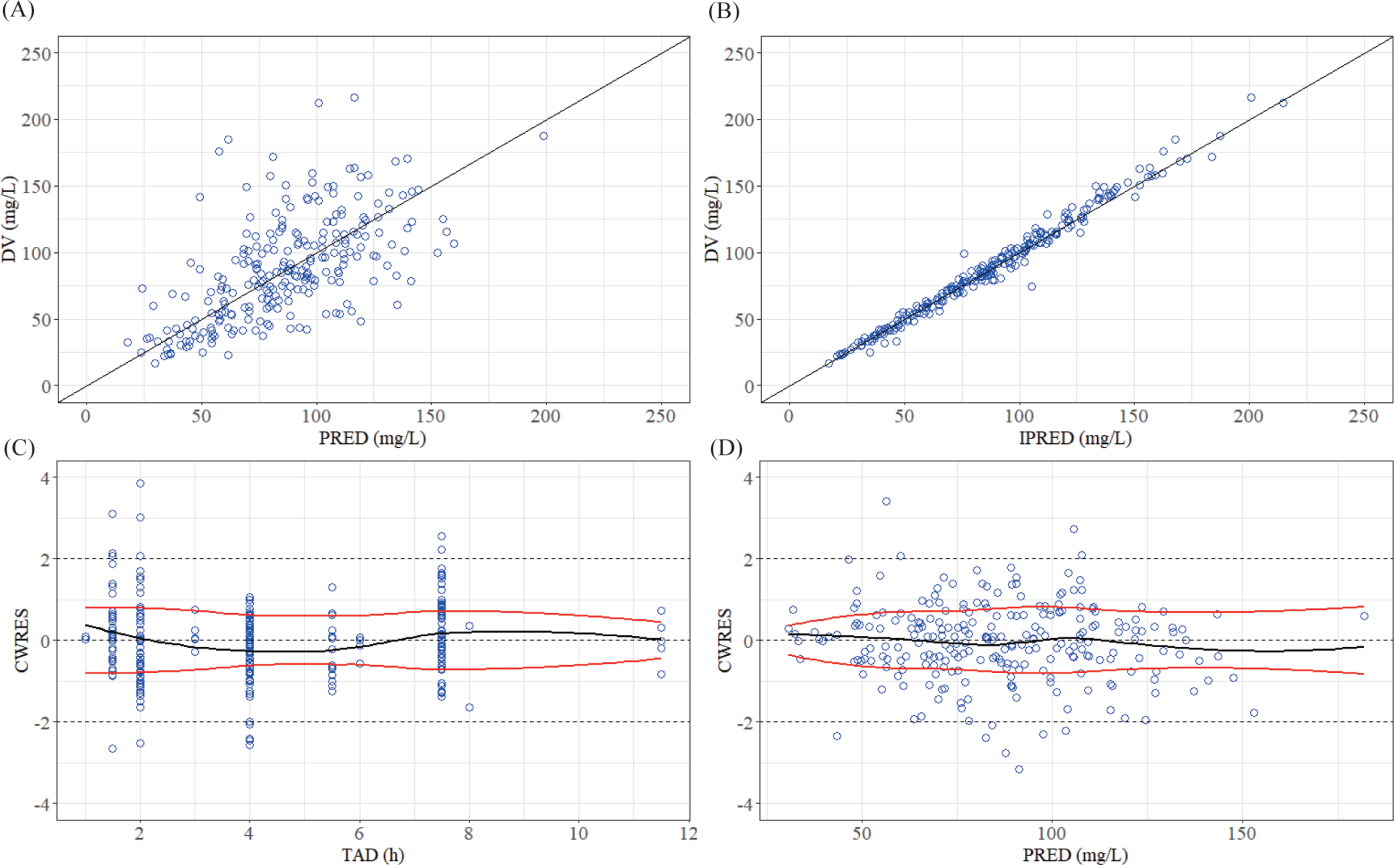
GOF plots of the SMX final model. (**A**) Observed concentration (DV) vs population-predicted concentration (PRED). (**B**) DV vs individual-predicted concentration (IPRED) (**C**) Conditional weighted residuals (CWRES) vs time after dose (TAD). (**D**) CWRES vs PRED. For parts A and B, the black line represents the locally weighted scatterplot smoothing (Loess) curve of the overall residual, and the red line represents the unilateral LOESS curve of the residual.

**Fig 2:**
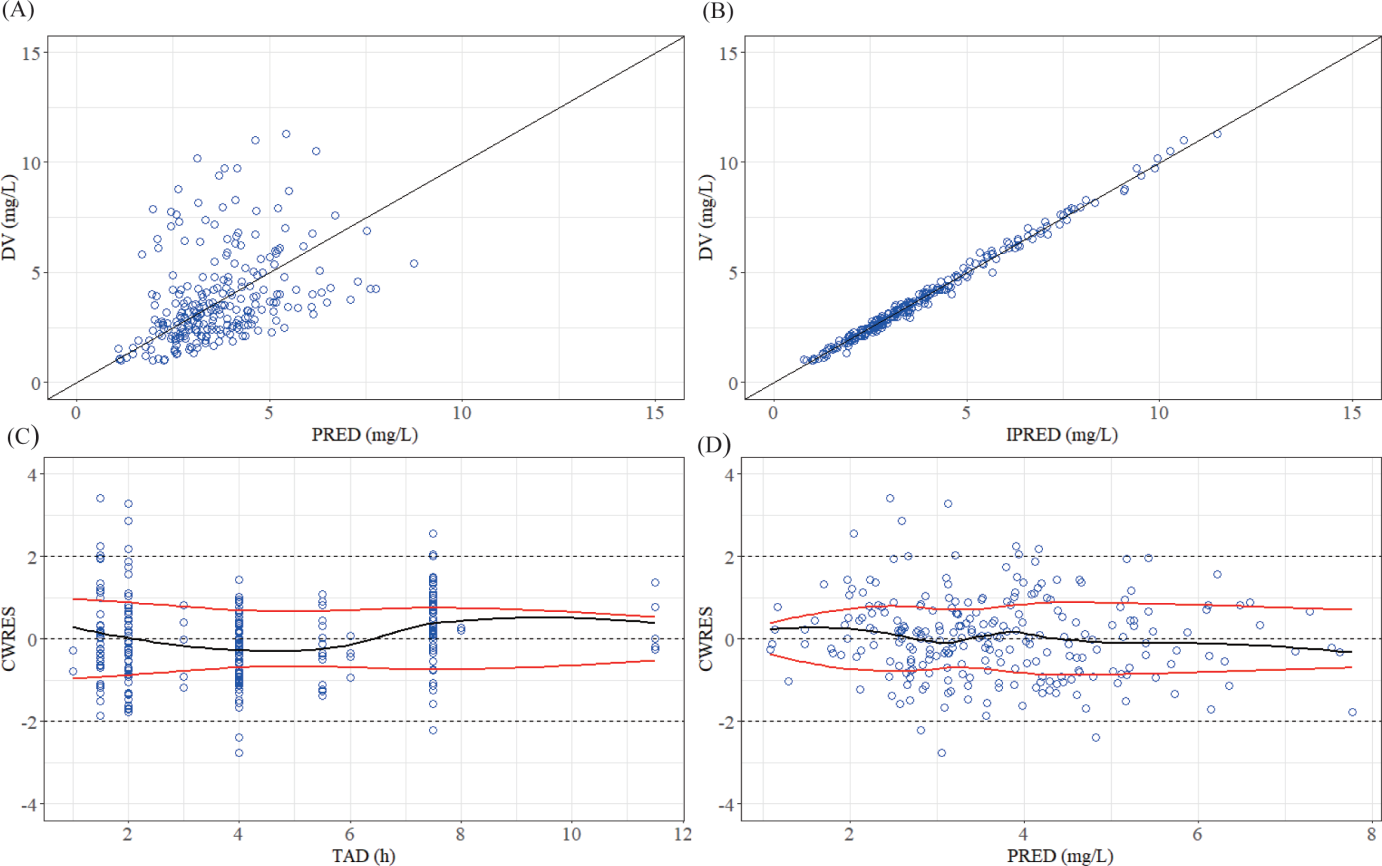
GOF plots of the TMP final model. (**A**) DV vs PRED. (**B**) DV vs IPRED. (**C**) CWRES vs TAD. (**D**) CWRES vs PRED.

**Fig 3:**
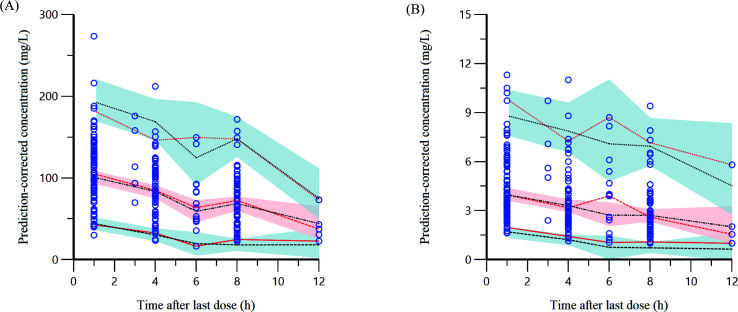
(**A**) Prediction-corrected visual predictive check of the simulation of the SMX final model. (**B**) Prediction-corrected visual predictive check of the simulation of the TMP final model. The prediction-corrected observed concentrations are illustrated as blue circles. Confidence intervals of the 5th, 50th, and 95th percentiles of the prediction-corrected observed data are represented by red lines. Confidence intervals of the 5th, 50th, and 95th percentiles of the prediction-corrected simulated data are represented by black lines. The shaded regions represent the 90% prediction intervals of the 5th, 50th, and 95th percentiles of prediction-corrected simulated data.

The GOF and pc-VPC plots, as illustrated in Fig. 1 to 3, demonstrate that both models provided an adequate description of the observed concentrations. The bootstrap results, summarized in Tables 5 and 6, indicate that the PopPK parameters of the final model exhibited good stability.

### Monte Carlo simulations

**Fig 4:**
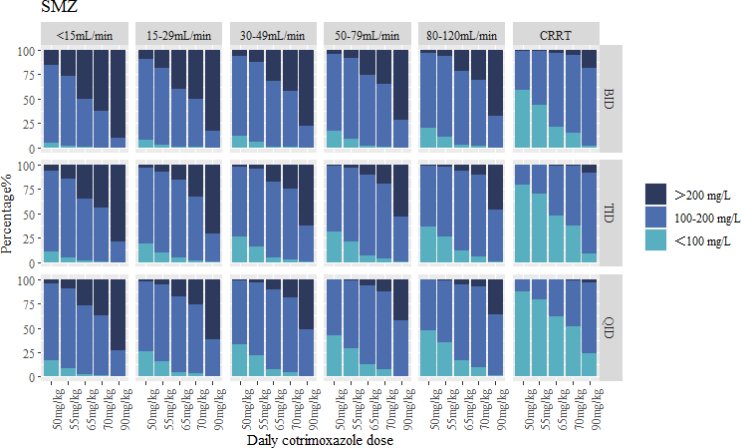
Target attainment of SMX for infections with PCP for different dosing regimens, different CrCL, or CRRT.

**Fig 5:**
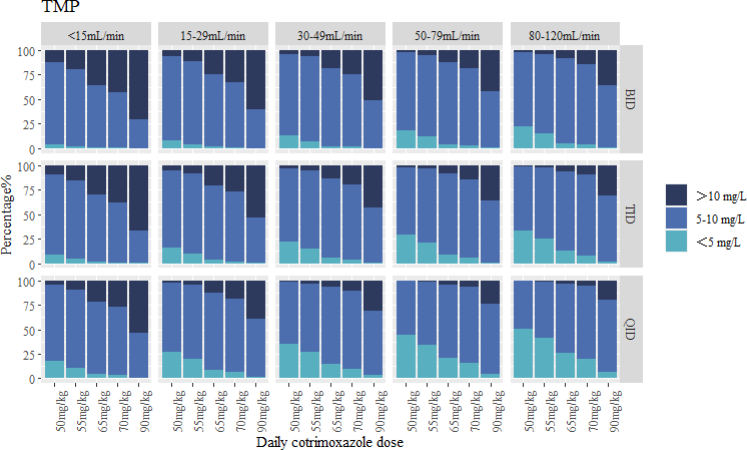
Target attainment of TMP for infections with PCP for different dosing regimens and different CrCLs.

The simulated results that were achievable within the incremental dosage regimen of patients treated with co-trimoxazole were simulated and stratified according to CrCL and CRRT (Fig. 4 and 5). As co-trimoxazole is a fixed-dose combination, the exposure of both SMX and TMP must be carefully considered. In patients with normal renal function (CrCL 80–120 mL/min), a TID 70 mg/kg/day dosing regimen maintained therapeutic concentrations for 84.9% of patients for SMX (5.6% below and 9.5% above the target range) and 82.6% of patients for TMP (7.7% below and 9.7% above the target range). In contrast, the guideline-recommended 90 mg/kg/day dose demonstrated elevated toxicity risks across all dosing intervals, with >20% of patients exhibiting supratherapeutic exposures.

Patients with diminished CrCL necessitated either dose reduction or increased dosing frequency to mitigate excessive exposure risks. In patients with CrCL of 50–79 mL/min, a TID 65 mg/kg/day regimen demonstrated therapeutic target attainment in 83.2% of patients for SMX (6.7% below and 10.1% above) and 82.4% for TMP (9.1% below and 8.5% above). For those with CrCL of 30–49 mL/min, a BID 55 mg/kg/day dosing regimen achieves SMX concentrations within the target range for 82.1% of patients (7.6% below and 10.3% above), while 86.5% of patients achieve TMP concentrations within the target range (6.9% below and 6.6% above). In cases of CrCL of 15–29 mL/min, a TID 55 mg/kg/day dosing regimen results in 82.4% of patients achieving SMX concentrations within the target range (10.0% below and 7.6% above) and 82.2% of patients achieving TMP concentrations within the target range (9.6% below and 8.2% above). For patients with CrCL of <15 mL/min, further dose reduction is required. A TID 50 mg/kg/day dosing regimen achieves SMX concentrations within the target range for 82.6% of patients (10.9% below and 6.5% above), while TMP concentrations remain within the target range for 81.3% of patients (9.5% below and 9.2% above).

In patients undergoing CRRT, SMX clearance was significantly increased. Under the TID 90 mg/kg/day dosing regimen, 82.6% of patients achieved SMX concentrations within the target range, with 8.9% below and 8.5% above the target. However, the model did not reveal a significant correlation between CRRT and TMP exposure; therefore, Monte Carlo simulations for TMP were not performed in this patient population.

Based on our population pharmacokinetic modeling and Monte Carlo simulations, we recommend a co-trimoxazole regimen of TID 70 mg/kg/day for patients with normal renal function (CrCL 80–120 mL/min). For patients requiring CRRT, an intensified regimen of TID 90 mg/kg/day is proposed to compensate for enhanced drug clearance. In renally impaired populations, individualized dosing adjustments stratified by creatinine clearance levels are essential, with specific regimens detailed in Table 7 to mitigate toxicity risks while maintaining therapeutic efficacy.

**TABLE 7:** Recommended dosing regimens stratified by renal function based on the study

Renal function^*a*^	Co-trimoxazole dose (mg/kg/day)	SMX (mg/L)			TMP (mg/L)		
		<100	100–200	>200	<5	5–10	>10
<15 mL/min	TID 50	10.9%	82.6%	6.5%	9.2%	81.3%	9.5%
15–29 mL/min	TID 55	10.0%	82.4%	7.6%	9.6%	82.2%	8.2%
30–49 mL/min	BID 55	7.6%	82.1%	10.3%	6.9%	86.5%	6.6%
50–79 mL/min	TID 65	6.7%	83.2%	10.1%	9.1%	82.4%	8.5%
80–120 mL/min	TID 70	5.6%	84.9%	9.5%	7.7%	82.6%	9.7%
CRRT	TID 90	8.9%	82.6%	8.5%	–^*b*^	–	–

^*a*^ Renal function was categorized according to CrCL thresholds and CRRT.

^*b*^ – are used to denote the ranges of CrCL values.

## DISCUSSION

In recent years, the clinical use of co-trimoxazole has increased alongside the rising incidence of PCP. However, research on its PopPK remains limited. This study represents the first PopPK analysis of intravenous SMX and TMP in PCP patients, incorporating Monte Carlo simulations for dose optimization. A one-compartment model effectively described the pharmacokinetics of SMX and TMP. Comprehensive covariate screening identified CrCL and CRRT as key factors influencing drug clearance in patients with renal dysfunction. These findings underscore the importance of adjusting SMX and TMP dosages based on renal function to ensure optimal drug exposure.

The final model estimated typical population CL values of 0.02 L/kg/h for SMX and 0.11 L/kg/h for TMP, aligning with previously reported ranges (SMX: 0.013–0.024 L/kg/h, TMP: 0.071–0.11L/kg/h ) (7, 26, 33, 34). Similarly, the typical apparent volumes of *V* were 0.32 L/kg for SMX and 2.22 L/kg for TMP, consistent with prior adult pharmacokinetic studies (SMX: 0.17–0.34 L/kg, TMP: 1.0–2.4 L/kg).

Current guidelines recommend a co-trimoxazole dosing regimen of 90–120 mg/kg/day, with mandatory dose reductions for patients with renal impairment (25). The FDA prescribing information contraindicates its use in patients with CrCL f <15 mL/min, recommends halved doses for CrCL of 15–30 mL/min, and two-thirds of the standard dose for CrCL of 30–50 mL/min (35, 36). Our study revealed that in patients with normal renal function (CrCL 80–120 mL/min), 35% and 20% exhibited supratherapeutic concentrations of SMX and TMP, respectively, when administered the guideline-recommended 90 mg/kg/day every 6 h. The BID 90 mg/kg/day regimen demonstrated substantially higher overexposure risks (67.3% SMX and 45.8% TMP). In contrast, the TID 70 mg/kg/day regimen achieved optimal therapeutic exposure with improved safety profiles. Dose reductions are mandatory for renally impaired patients, while those undergoing CRRT require intensified dosing regimens. Contrary to previous simulations by Leegwater et al. (showing no regimens achieved adequate SMX exposure, with only 65% target attainment) (37), our optimized regimen achieved a probability of target attainment exceeding 80% for both SMX and TMP. Furthermore, increased dosing frequency at equivalent total daily doses demonstrated a moderate reduction in adverse event risks.

Co-trimoxazole is available in both oral and intravenous formulations, with the option to switch between routes during treatment. While oral bioavailability approaches 100%, intravenous administration achieves higher peak plasma concentrations more rapidly, making it preferable for severe infections such as PCP and meningitis (33). Intravenous administration ensures rapid attainment of therapeutic concentrations, reduces mortality, and maintains a synergistic SMX-to-TMP ratio of 5:1, enhancing antibacterial efficacy (23, 26). In contrast, oral absorption is subject to food effects and gastrointestinal variability, potentially leading to subtherapeutic levels in critically ill patients (38). However, oral administration remains safe and effective for PCP prophylaxis at a recommended dose of TID 480 mg (39, 40).

This study explored the effects of liver function and *NAT2* and *CYP2CP* genotypes on SMX pharmacokinetics. Previous pharmacokinetic studies have shown that 40% of SMX is metabolized in the liver to inactive N-acetylated compounds, with NAT2 and *CYP2C9* enzymes playing a major role in this process (9). Our pharmacogenomic analysis demonstrates limited influence of *CYP2C9* and *NAT2* genetic variants on SMX CL. In a study investigating co-trimoxazole-induced hepatotoxicity among Han Chinese individuals, *CYP2C9* polymorphisms demonstrated no significant association with hepatic adverse events. This predisposition is hypothesized to arise from compromised detoxification capacity leading to reactive metabolite accumulation (41). This association may stem from impaired detoxification capacity leading to bioactivated metabolite accumulation. Additionally, the Child-Pugh classification did not significantly affect SMX pharmacokinetics, although severe liver dysfunction may impair SMX metabolism, leading to elevated concentrations. Some studies suggested that impaired liver function did not seem to affect peak SMX concentrations (42, 43). However, a study by Klinker et al. on SMX blood concentration measurement indicated that severe liver dysfunction impairs the metabolism of SMX, leading to higher SMX concentrations in patients (8). Furthermore, there is a correlation between ALB levels and SMX CL, indicating that the liver plays a role in the metabolic elimination of SMX to some extent (44).

Both SMX and TMP undergo renal elimination, making renal function a critical determinant of their CL (11). Covariate analysis identified CrCL as a significant covariate in both pharmacokinetic models. Patients with severe renal impairment exhibited dose-dependent drug exposure, underscoring the necessity for careful dosing adjustments. While the half-life of SMX is only marginally prolonged in patients with renal failure, its primary metabolite, N-acetyl sulfamethoxazole, is predominantly renally excreted (45). The accumulation of N-acetyl sulfamethoxazole has nephrotoxic potential, which may exacerbate pre-existing renal impairment (7). Lau and Young observed that patients who were unresponsive to co-trimoxazole therapy had lower peak TMP concentrations compared to rapid responders, emphasizing the importance of appropriate loading doses to achieve prompt therapeutic effects (46). Therefore, when selecting an optimal dosage regimen, it is essential to balance the need for early effective exposure with the risk of toxicity. Additionally, age significantly influences renal function. Pediatric pharmacokinetic studies demonstrate that renal function progressively matures with age, correlating with improved treatment safety (44). A comparative study between young and elderly subjects revealed a significantly reduced mean renal clearance of TMP in elderly individuals compared to their younger counterparts (47). Consequently, TDM is strongly recommended for patients with severe renal impairment or advanced age.

CRRT was included as a covariate in the SMX model but not in the TMP model. Curkovic et al. reported that TMP CL falls within the lower range of renal clearance observed in patients with normal renal function, while SMX CL significantly exceeds normal renal clearance in patients undergoing CRRT (48). Consequently, patients receiving CRRT require higher doses of SMX, likely due to the absence of tubular reabsorption of SMX in these individuals (48, 49). SMX is directly eliminated from the body through ultrafiltration, resulting in increased clearance during CRRT. In contrast, TMP has a large volume of distribution, meaning that the drug concentration in plasma represents only a small fraction of the total amount in the body (4). Additionally, TMP exhibits approximately 60% protein binding, further limiting its removal by CRRT (50). As a result, TMP dosage adjustments may not be necessary in these patients, whereas higher doses of SMX are required to ensure adequate drug exposure.

This study has several limitations. First, the limited sample size may constrain the establishment of robust associations between co-trimoxazole exposure and nephrotoxicity. Second, the small number of patients receiving CRRT precluded an assessment of the impact of different CRRT settings on pharmacokinetics. Future prospective studies with larger cohorts are needed to validate these findings and refine dosing recommendations. Finally, we did not account for concomitant administration of other antibiotics and their influence on the treatment efficacy or nephrotoxicity.

### Conclusion

This study successfully established PopPK models for intravenous SMX and TMP. CrCL was identified as a significant covariate influencing the CL of both SMX and TMP, whereas CRRT significantly affected only SMX CL. These findings suggest that patients with renal impairment require dose adjustments. Our study provides a scientific basis for individualized co-trimoxazole dosing strategies in patients receiving intravenous administration.

## References

[1] Howe RA, Spencer RC. 1996. Cotrimoxazole. Rationale for re-examining its indications for use. Drug Saf 14:213–218. doi:10.2165/00002018-199614040-00001 8713689

[2] Wormser GP, Keusch GT, Heel RC. 1982. Co-trimoxazole (trimethoprim-sulfamethoxazole): an updated review of its antibacterial activity and clinical efficacy. Drugs (Abingdon Engl) 24:459–518. doi:10.2165/00003495-198224060-00002 6759092

[3] Weyant RB, Kabbani D, Doucette K, Lau C, Cervera C. 2021. Pneumocystis jirovecii: a review with a focus on prevention and treatment. Expert Opin Pharmacother 22:1579–1592. doi:10.1080/14656566.2021.1915989 33870843

[4] Vouloumanou EK, Karageorgopoulos DE, Rafailidis PI, Michalopoulos A, Falagas ME. 2011. Trimethoprim/sulfametrole: evaluation of the available clinical and pharmacokinetic/pharmacodynamic evidence. Int J Antimicrob Agents 38:197–216. doi:10.1016/j.ijantimicag.2011.04.016 21741802

[5] Guidi M, Csajka C, Buclin T. 2022. Parametric approaches in population pharmacokinetics. J Clin Pharmacol 62:125–141. doi:10.1002/jcph.1633 33103774

[6] Lepak AJ, Andes DR. 2014. Antifungal pharmacokinetics and pharmacodynamics. Cold Spring Harb Perspect Med 5:a019653. doi:10.1101/cshperspect.a019653 25384765 PMC4448584

[7] Siber GR, Gorham CC, Ericson JF, Smith AL. 1982. Pharmacokinetics of intravenous trimethoprim-sulfamethoxazole in children and adults with normal and impaired renal function. Rev Infect Dis 4:566–578. doi:10.1093/clinids/4.2.566 6981173

[8] Klinker H, Langmann P, Zilly M, Richter E. 1998. Drug monitoring during the treatment of AIDS-associated Pneumocystis carinii pneumonia with trimethoprim-sulfamethoxazole. J Clin Pharm Ther 23:149–154. doi:10.1046/j.1365-2710.1998.00152.x 9786102

[9] Soejima M, Sugiura T, Kawaguchi Y, Kawamoto M, Katsumata Y, Takagi K, Nakajima A, Mitamura T, Mimori A, Hara M, Kamatani N. 2007. Association of the diplotype configuration at the N-acetyltransferase 2 gene with adverse events with co-trimoxazole in Japanese patients with systemic lupus erythematosus. Arthritis Res Ther 9:R23. doi:10.1186/ar2134 17335581 PMC1906798

[10] Tsvetkova N, Harizanov R, Rainova I, Ivanova A, Yancheva-Petrova N. 2023. Molecular analysis of dihydropteroate synthase gene mutations in Pneumocystis jirovecii isolates among Bulgarian patients with Pneumocystis pneumonia. Int J Mol Sci 24:16927. doi:10.3390/ijms242316927 38069248 PMC10707730

[11] Trollfors B, Wahl M, Alestig K. 1980. Co-trimoxazole, creatinine and renal function. J Infect 2:221–226. doi:10.1016/s0163-4453(80)90626-x 6821086

[12] Singlas E, Colin JN, Rottembourg J, Meessen JP, de Martin A, Legrain M, Simon P. 1982. Pharmacokinetics of sulfamethoxazole--trimethoprim combination during chronic peritoneal dialysis: effect of peritonitis. Eur J Clin Pharmacol 21:409–415. doi:10.1007/BF00542328 7075646

[13] Blaser J, Joos B, Opravil M, Lüthy R. 1993. Variability of serum concentrations of trimethoprim and sulfamethoxazole during high dose therapy. Infection 21:206–209. doi:10.1007/BF01728888 8225622

[14] Hughes WT, Feldman S, Chaudhary SC, Ossi MJ, Cox F, Sanyal SK. 1978. Comparison of pentamidine isethionate and trimethoprim-sulfamethoxazole in the treatment of Pneumocystis carinii pneumonia. J Pediatr 92:285–291. doi:10.1016/s0022-3476(78)80028-6 304478

[15] Shimizu Y, Hirai T, Ogawa Y, Yamada C, Kobayashi E. 2022. Characteristics of risk factors for acute kidney injury among inpatients administered sulfamethoxazole/trimethoprim: a retrospective observational study. J Pharm Health Care Sci 8:20. doi:10.1186/s40780-022-00251-0 35909129 PMC9341082

[16] Shann F. 1984. Co-trimoxazole toxicity. Lancet 2:1477. doi:10.1016/s0140-6736(84)91677-5 6151091

[17] Alappan R, Perazella MA, Buller GK. 1996. Hyperkalemia in hospitalized patients treated with trimethoprim-sulfamethoxazole. Ann Intern Med 124:316–320. doi:10.7326/0003-4819-124-3-199602010-00006 8554227

[18] Fishman JA. 1998. Treatment of infection due to Pneumocystis carinii. Antimicrob Agents Chemother 42:1309–1314. doi:10.1128/AAC.42.6.1309 9624465 PMC105593

[19] Abdul-Aziz MH, Alffenaar J-WC, Bassetti M, Bracht H, Dimopoulos G, Marriott D, Neely MN, Paiva J-A, Pea F, Sjovall F, Timsit JF, Udy AA, Wicha SG, Zeitlinger M, De Waele JJ, Roberts JA, the Infection Section of European Society of Intensive Care Medicine (ESICM), Pharmacokinetic/pharmacodynamic and Critically Ill Patient Study Groups of European Society of Clinical Microbiology and Infectious Diseases (ESCMID), Infectious Diseases Group of International Association of Therapeutic Drug Monitoring and Clinical Toxicology (IATDMCT), Infections in the ICU and Sepsis Working Group of International Society of Antimicrobial Chemotherapy (ISAC). 2020. Antimicrobial therapeutic drug monitoring in critically ill adult patients: a position paper#. Intensive Care Med 46:1127–1153. doi:10.1007/s00134-020-06050-1 32383061 PMC7223855

[20] Brown GR. 2014. Cotrimoxazole - optimal dosing in the critically ill. Ann Intensive Care 4:13. doi:10.1186/2110-5820-4-13 24910807 PMC4031607

[21] Rifkind D, Faris TD, Hill RB Jr. 1966. Pneumocystis carinii pneumonia. Studies on the diagnosis and treatment. Ann Intern Med 65:943–956. doi:10.7326/0003-4819-65-5-943 5332224

[22] Hughes WT, Feldman S, Sanyal SK. 1975. Treatment of Pneumocystis carinii pneumonitis with trimethoprim-sulfamethoxazole. Can Med Assoc J 112:47–50. 1079469 PMC1956451

[23] Joos B, Blaser J, Opravil M, Chave JP, Lüthy R. 1995. Monitoring of co-trimoxazole concentrations in serum during treatment of pneumocystis carinii pneumonia. Antimicrob Agents Chemother 39:2661–2666. doi:10.1128/AAC.39.12.2661 8592998 PMC163008

[24] Schulz M, Schmoldt A, Andresen-Streichert H, Iwersen-Bergmann S. 2020. Revisited: therapeutic and toxic blood concentrations of more than 1100 drugs and other xenobiotics. Crit Care 24:195. doi:10.1186/s13054-020-02915-5 32375836 PMC7201985

[25] Maschmeyer G, Helweg-Larsen J, Pagano L, Robin C, Cordonnier C, Schellongowski P, 6th European Conference on Infections in Leukemia (ECIL-6), a joint venture of The European Group for Blood and Marrow Transplantation (EBMT), The European Organization for Research and Treatment of Cancer (EORTC), the International Immunocompromised Host Society (ICHS) and The European LeukemiaNet (ELN). 2016. ECIL guidelines for treatment of Pneumocystis jirovecii pneumonia in non-HIV-infected haematology patients. J Antimicrob Chemother 71:2405–2413. doi:10.1093/jac/dkw158 27550993

[26] Gleckman R, Gantz NM, Joubert DW. 1981. Intravenous sulfamethoxazole-trimethoprim: pharmacokinetics, therapeutic indications, and adverse reactions. Pharmacotherapy 1:206–211. doi:10.1002/j.1875-9114.1981.tb02542.x 6985449

[27] Pierrat A, Gravier E, Saunders C, Caira MV, Aït-Djafer Z, Legras B, Mallié JP. 2003. Predicting GFR in children and adults: a comparison of the Cockcroft-Gault, Schwartz, and modification of diet in renal disease formulas. Kidney Int 64:1425–1436. doi:10.1046/j.1523-1755.2003.00208.x 12969162

[28] Bellomo R, Ronco C, Kellum JA, Mehta RL, Palevsky P. 2004. Acute renal failure - definition, outcome measures, animal models, fluid therapy and information technology needs: the Second International Consensus Conference of the Acute Dialysis Quality Initiative (ADQI) Group. Crit Care 8:R204–12. doi:10.1186/cc2872 15312219 PMC522841

[29] Dijkstra JA, Alsaad NS, Hateren K van, Greijdanus B, Touw DJ, Alffenaar J-WC. 2015. Quantification of co-trimoxazole in serum and plasma using MS/MS. Bioanalysis 7:2741–2749. doi:10.4155/bio.15.188 26566213

[30] Kagaya H, Miura M, Niioka T, Saito M, Numakura K, Habuchi T, Satoh S. 2012. Influence of NAT2 polymorphisms on sulfamethoxazole pharmacokinetics in renal transplant recipients. Antimicrob Agents Chemother 56:825–829. doi:10.1128/AAC.05037-11 22106207 PMC3264276

[31] Theken KN, Lee CR, Gong L, Caudle KE, Formea CM, Gaedigk A, Klein TE, Agúndez JAG, Grosser T. 2020. Clinical Pharmacogenetics Implementation Consortium Guideline (CPIC) for CYP2C9 and nonsteroidal anti-inflammatory drugs. Clin Pharmacol Ther 108:191–200. doi:10.1002/cpt.1830 32189324 PMC8080882

[32] Stevens RC, Laizure SC, Sanders PL, Stein DS. 1993. Multiple-dose pharmacokinetics of 12 milligrams of trimethoprim and 60 milligrams of sulfamethoxazole per kilogram of body weight per day in healthy volunteers. Antimicrob Agents Chemother 37:448–452. doi:10.1128/AAC.37.3.448 8460913 PMC187691

[33] Chin TW, Vandenbroucke A, Fong IW. 1995. Pharmacokinetics of trimethoprim-sulfamethoxazole in critically ill and non-critically ill AIDS patients. Antimicrob Agents Chemother 39:28–33. doi:10.1128/AAC.39.1.28 7695325 PMC162479

[34] Patel RB, Welling PG. 1980. Clinical pharmacokinetics of co-trimoxazole (trimethoprim-sulphamethoxazole). Clin Pharmacokinet 5:405–423. doi:10.2165/00003088-198005050-00001 7408366

[35] Gilbert D, Chambers H, Eliopoulos G. 2020. The Sanford guide to antimicrobial therapy 2020. 50th ed. Antimicrobial Therapy. Inc, Sperryville.

[36] Wilson JW, Estes LL. 2018. Mayo clinic antimicrobial therapy: quick guide. Oxford University Press.

[37] Leegwater E, Baidjoe L, Wilms EB, Visser LG, Touw DJ, de Winter BCM, de Boer MGJ, van Paassen J, van den Berg CHSB, van Prehn J, van Gelder T, Moes DJAR. 2025. Population pharmacokinetics of trimethoprim/sulfamethoxazole: dosage optimization for patients with renal insufficiency or receiving continuous renal replacement therapy. Clin Pharmacol Ther 117:184–192. doi:10.1002/cpt.3421 39148353 PMC11652823

[38] Winston DJ, Lau WK, Gale RP, Young LS. 1980. Trimethoprim-sulfamethoxazole for the treatment of Pneumocystis carinii pneumonia. Ann Intern Med 92:762–769. doi:10.7326/0003-4819-92-6-762 6966901

[39] Nagai T, Matsui H, Fujioka H, Homma Y, Otsuki A, Ito H, Ohmura S, Miyamoto T, Shichi D, Tomohisa W, Otsuka Y, Nakashima K. 2024. Low-dose vs conventional-dose trimethoprim-sulfamethoxazole treatment for Pneumocystis pneumonia in patients not infected with HIV: a Multicenter, Retrospective Observational Cohort Study. Chest 165:58–67. doi:10.1016/j.chest.2023.08.009 37574166

[40] McDonald EG, Afshar A, Assiri B, Boyles T, Hsu JM, Khuong N, Prosty C, So M, Sohani ZN, Butler-Laporte G, Lee TC. 2024. Pneumocystis jirovecii pneumonia in people living with HIV: a review. Clin Microbiol Rev 37:e0010122. doi:10.1128/cmr.00101-22 38235979 PMC10938896

[41] Huang YS, Tseng SY, Chang TE, Perng CL, Huang YH. 2021. Sulfamethoxazole-trimethoprim-induced liver injury and genetic polymorphisms of NAT2 and CYP2C9 in Taiwan. Pharmacogenet Genomics 31:200–206. doi:10.1097/FPC.0000000000000441 34149005

[42] Bergan T, Brodwall EK. 1972. Human pharmacokinetics of a sulfamethoxazole-trimethoprim combination. Acta Med Scand 192:483–492. doi:10.1111/j.0954-6820.1972.tb04852.x 4640694

[43] Lee BL, Ileana M, Benowitz NL, Peyton J 3rd III, Wofsy CB, John MV. 1989. Dapsone, trimethoprim, and sulfamethoxazole plasma levels during treatment of Pneumocystis pneumonia in patients with the acquired immunodeficiency syndrome (AIDS). Evidence of drug interactions. Ann Intern Med 110:606–611. doi:10.7326/0003-4819-110-8-606 2784648

[44] Autmizguine J, Melloni C, Hornik CP, Dallefeld S, Harper B, Yogev R, Sullivan JE, Atz AM, Al-Uzri A, Mendley S, Poindexter B, Mitchell J, Lewandowski A, Delmore P, Cohen-Wolkowiez M, Gonzalez D, the Pediatric Trials Network Steering Committee. 2018. Population pharmacokinetics of trimethoprim-sulfamethoxazole in infants and children. Antimicrob Agents Chemother 62:e01813-17. doi:10.1128/AAC.01813-17 29084742 PMC5740321

[45] Craig WA, Kunin CM. 1973. Trimethoprim-sulfamethoxazole: pharmacodynamic effects of urinary pH and impaired renal function. Studies in humans. Ann Intern Med 78:491–497. doi:10.7326/0003-4819-78-4-491 4571565

[46] Lau WK, Young LS. 1976. Trimethoprim-sulfamethoxazole treatment of Pneumocystis carinii pneumonia in adults. N Engl J Med 295:716–718. doi:10.1056/NEJM197609232951308 1085413

[47] Varoquaux O, Lajoie D, Gobert C, Cordonnier P, Ducreuzet C, Pays M, Advenier C. 1985. Pharmacokinetics of the trimethoprim-sulphamethoxazole combination in the elderly. Br J Clin Pharmacol 20:575–581. doi:10.1111/j.1365-2125.1985.tb05114.x 3879182 PMC1400827

[48] Curkovic I, Lüthi B, Franzen D, Ceschi A, Rudiger A, Corti N. 2010. Trimethoprim/Sulfamethoxazole pharmacokinetics in two patients undergoing continuous venovenous hemodiafiltration. Ann Pharmacother 44:1669–1672. doi:10.1345/aph.1P160 20823279

[49] Clajus C, Kühn-Velten WN, Schmidt JJ, Lorenzen JM, Pietsch D, Beutel G, Kielstein JT. 2013. Cotrimoxazole plasma levels, dialyzer clearance and total removal by extended dialysis in a patient with acute kidney injury: risk of under-dosing using current dosing recommendations. BMC Pharmacol Toxicol 14:19. doi:10.1186/2050-6511-14-19 23551893 PMC3626772

[50] Roberts DM, Roberts JA, Roberts MS, Liu X, Nair P, Cole L, Lipman J, Bellomo R, RENAL Replacement Therapy Study Investigators. 2012. Variability of antibiotic concentrations in critically ill patients receiving continuous renal replacement therapy: a multicentre pharmacokinetic study. Crit Care Med 40:1523–1528. doi:10.1097/CCM.0b013e318241e553 22511133

